# Retraction: Application of edge computing and GIS in ecological water requirement prediction and optimal allocation of water resources in irrigation area

**DOI:** 10.1371/journal.pone.0305356

**Published:** 2024-06-06

**Authors:** The PLOS ONE Editors

The *PLOS ONE* Editors identified this article [1] as one of a series of submissions for which we have concerns about peer review integrity and similarities across articles. These concerns call into question the validity and provenance of the reported results. We regret that the issues were not identified prior to the article’s publication.

During post-publication discussions about these concerns, Rengui Jiang (RJ) informed PLOS that they did not contribute to this article and were not aware that they had been listed as a co-author. The corresponding author (YL) subsequently stated that they prepared and submitted the manuscript alone, and all other authors did not contribute and were not aware that they had been listed as co-authors.

In light of the above concerns, the *PLOS ONE* Editors retract this article.

YL did not agree with the retraction. RJ responded but neither agreed nor disagreed with the retraction. JX and DY either did not respond directly or could not be reached.
